# Lower mortality of COVID-19 by early recognition and intervention: experience from Jiangsu Province

**DOI:** 10.1186/s13613-020-00650-2

**Published:** 2020-03-18

**Authors:** Qin Sun, Haibo Qiu, Mao Huang, Yi Yang

**Affiliations:** 1 Department of Critical Care Medicine, Zhongda Hospital, School of Medicine, Southeast University, No. 87, Dingjiaqiao Road, Gulou District, Nanjing, 210009 People’s Republic of China; 2 Department of Respiratory and Critical Care Medicine, The First Affiliated Hospital of Nanjing Medical University, 300 Guangzhou Road, Nanjing, 210029 People’s Republic of China

A cluster of patients of novel coronavirus pneumonia (NCP) have been identified in Wuhan in December 2019 and soon this virus spread at a tremendous rate which swept through the whole China and more than 93 countries and regions around the world [1, 2]. This emerging, rapidly evolving situation has threatened the health of all mankind and WHO has raised COVID-19 risk to “very high” at global level.

Up to now, 80,859 cases were confirmed, among which 10–15% patients were critically ill and 3100 (3.83%) died in China. The large number of transmission population between Jiangsu and Hubei provinces led to the infinite burden in controlling the COVID-19 epidemic in Jiangsu Province [3, 4]. By 24:00 on March 7, a total of 631 confirmed cases of NCP were reported with a portion of critically ill patients whose ages ranged from 9 months to 96 years old. A total of 610 cases have been discharged from hospital, and the cure rate of confirmed cases in our province has reached 96.67%, which is far exceeding that of national data [5–8]. Since the outcome of NCP patients in Jiangsu was much better than that in Hubei where the mortality of NCP patients was nearly 4.34%, we retrospectively summarized our therapeutic process and figured out that critical care-dominated treatment patterns might be the core in reducing mortality.

1. Early recognition of high-risk and critically ill patients

Since the severity of disease is closely related to the prognosis, the basic and essential strategies to improve outcomes that we should adhere to remain the early detection of high-risk and critically ill patients [9, 10]. During the clinical work in Jiangsu Province, critical care was shifted forward and early screening was measured. All NCP patients were screened twice every day and respiratory rate (RR), heart rate (HR), SpO2 (room air) were monitored regularly. Once SpO_2_ < 93%, RR > 30/min, HR > 120/min or any signs of organ failure were observed, patients would be transferred to intensive care unit (ICU) and ICU physicians and nurses would take over their treatment.

From our data of more than 600 NCP patients in Jiangsu Province, age, lymphocyte count, oxygen supplementation and aggressive pulmonary radiographic infiltrations are independent risk factors for NCP progressing to a critical condition. We established an early warning system combining these four factors to identify high-risk patients and then kept them under continuous close monitoring. The sensitivity of this warning system was 0.955 (95% CI [0.772–0.999]), the specificity was 0.899 (95% CI [0.863–0.928]) and the area under ROC curve was 0.962 (data unpublished). Our retrospective analysis of cases in Jiangsu Province proved a good consistency between early screening of SpO_2_, RR, HR and early warning model. Therefore, a flowchart integrating early warning model and early screening procedure is recommended for high-risk patients recognition and all patients’ screening to make it possible for early intervention (Fig. 1).

**Fig. 1:**
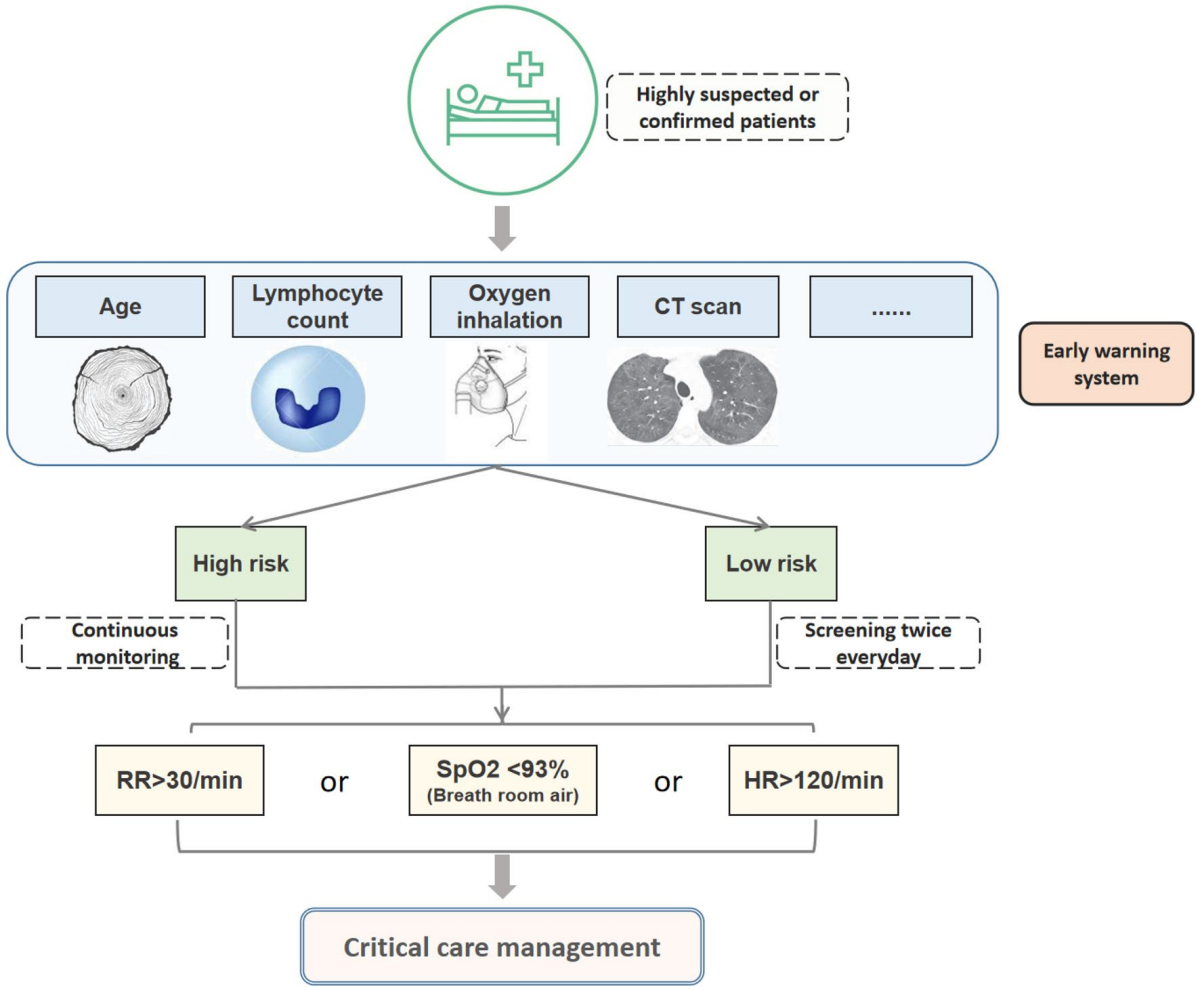
Early warning system and screening procedures for NCP patients

1. Early intervention guided by intensivists

Since there have been no effective antiviral treatments for COVID-19 [7, 8], the vital way to reduce mortality is early and strong intervention to prevent the progression of disease. During the treatment of Jiangsu NCP patients, three points which showed valid evidence in reversing the disease and preventing tracheal intubation rate were summarized.

(1) For patients with ARDS or pulmonary extensive effusion in CT scan, high-flow nasal cannula oxygen therapy (HFNC) or non-invasive mechanical ventilation (NIV) was used to maintain positive end expiratory pressure (PEEP) to prevent alveolar collapse even if some of these patients did not have refractory hypoxemia. (2) Restrictive fluid resuscitation under the premise of adequate tissue perfusion is performed to relieve pulmonary edema. (3) Although previous study proved prone position’s benefit in moderate-to-severe ARDS patients with invasive mechanical ventilation (IMV) [11], we attempted awake prone position in NCP patients which showed significant effects in improving oxygenation and pulmonary heterogeneity (Fig. 2). With all these measurements, although the rate of critically ill patients in Jiangsu had reached 10%, the IMV rate of Jiangsu was kept under 1%, which was significantly lower than our previous survey about ARDS patients [12].

**Fig. 2:**
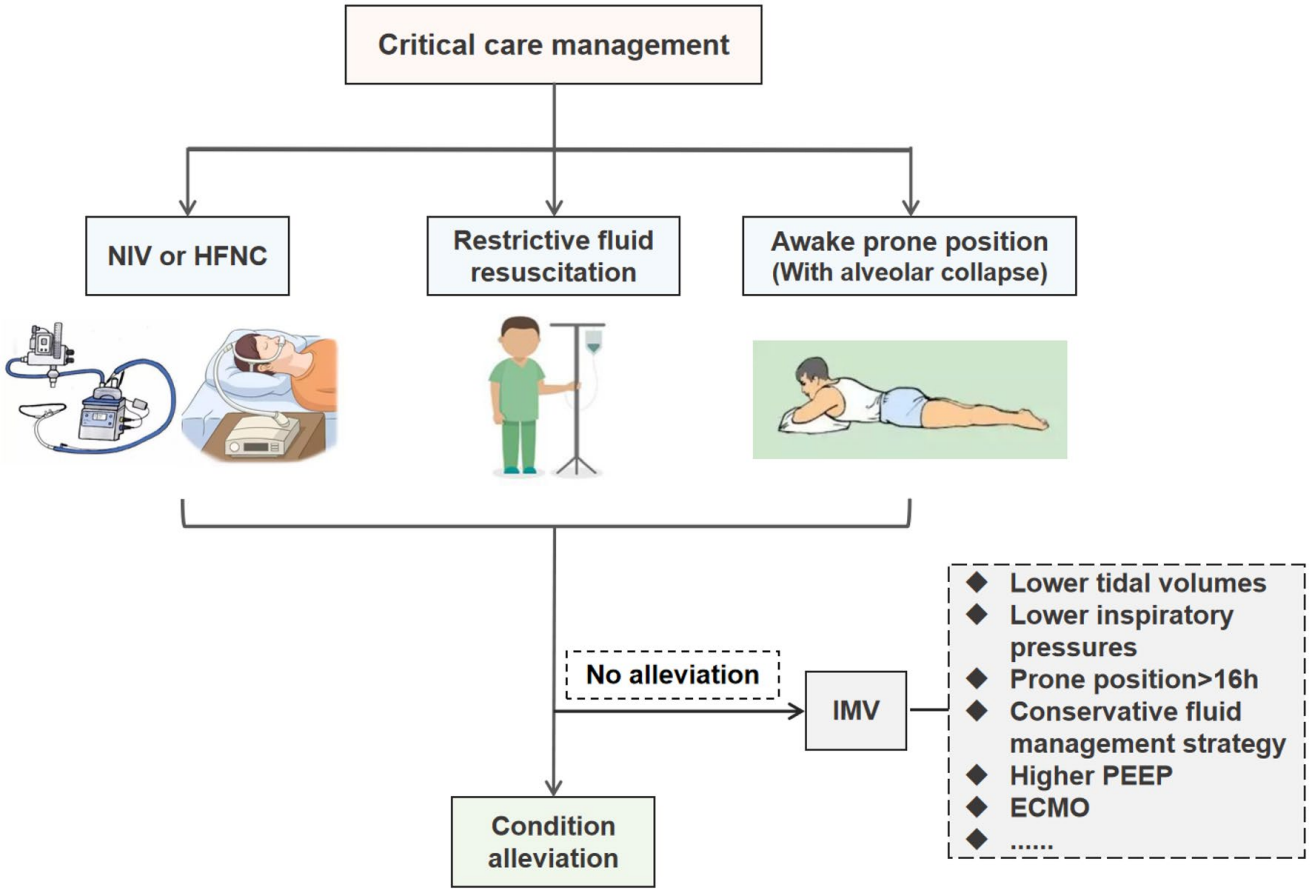
Early intervention for patients with critical condition

1. Clinical experts-guided hierarchical management strategy

At the outset of epidemic situation, a clinical experts-guided, multidisciplinary, province-wide hierarchical management group was established to provide medical guidance for all NCP patients [13]. The members of this panel are mainly critical care specialists and respiratory specialists from tertiary hospitals. Jiangsu Province is divided into five regions according to geographical position and each leader takes responsibilities for a specific region so that problems can be solved layer-by-layer. This kind of regional responsibility, timely feedback communication management makes it possible for effective medical interventions (Fig. 3).

**Fig. 3:**
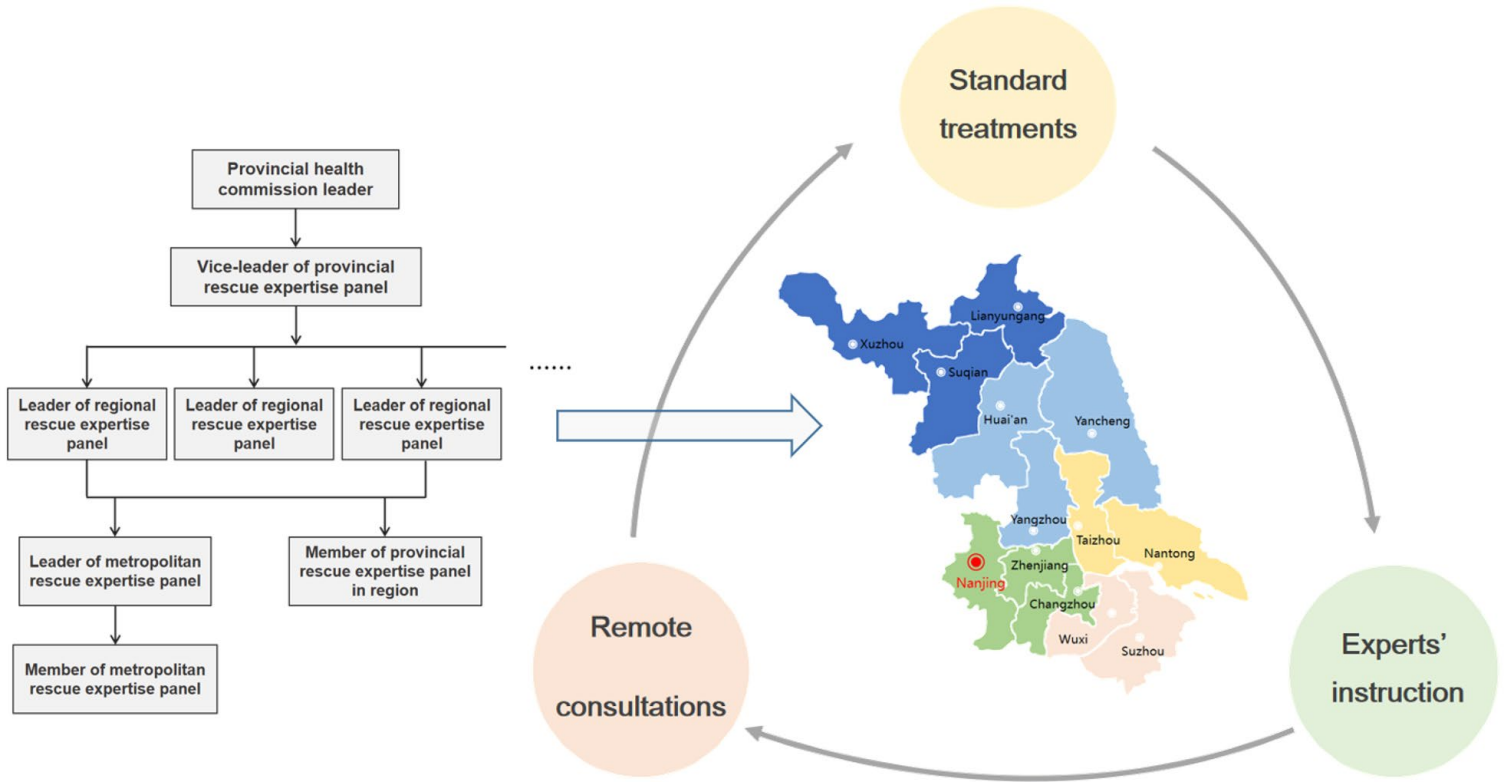
Organization chart of hierarchical management strategy

1. Rational allocation of materials and human resources

Health authorities attached great importance to epidemic and deployed disease prevention and control measures effectively [14, 15]. All kinds of resources, including frontline medical staff and medical protective materials, were mobilized and deployed uniformly to guarantee patients’ medical care. 234 clinical staff invested in NCP patients’ treatment and care, and 3500 clinical staff were reserved for unexpected needs. Adequate material and human resources are important cornerstones for controlling this epidemic.

Since the outbreak of COVID-19, Jiangsu takes effective measures to curb the spread of the virus and gives normative treatments for infected patients, which shows significant disease control and treatment effects. From our experience, early screening of critically ill patients and critical care-guided early intervention are prominent in reducing NCP patients’ mortality. At this critical moment in the global outbreak of NCP, we hope our valid management and treatment bundles can help us achieve the victory in the battle against COVID-19.

## Data Availability

Not applicable.
